# Cerebral Oxygenation During Transition and Amplitude‐Integrated Electroencephalography Signals: An Ancillary Study to the COSGOD‐III Trial

**DOI:** 10.1111/apa.70190

**Published:** 2025-06-27

**Authors:** Christina Schreiner, Katharina Goeral, Marlene Hammerl, Katrin Klebermass‐Schrehof, Alexander Avian, Vera Neubauer, Ursula Kiechl‐Kohlendorfer, Daniel Pfurtscheller, Elke Griesmaier, Gerhard Pichler

**Affiliations:** ^1^ Department of Paediatrics II (Neonatology) Medical University of Innsbruck Innsbruck Austria; ^2^ Department of Paediatrics and Adolescent Medicine, Division of Neonatology, Paediatric Intensive Care and Neuropaediatrics, Comprehensive Centre for Paediatrics Medical University of Vienna Vienna Austria; ^3^ Institute for Medical Informatics, Statistics and Documentation Medical University of Graz Graz Austria; ^4^ Division of Neonatology, Department of Paediatrics and Adolescent Medicine Medical University of Graz Graz Austria

**Keywords:** amplitude‐integrated electroencephalography, electrocortical activity, NIRS, preterm infant, total maturation score

## Abstract

**Aim:**

The COSGOD III trial was designed to guide oxygen delivery by cerebral near infrared spectroscopy (NIRS) in preterm neonates during the immediate transition after birth and showed a non‐significant increase of 4.3% in survival without cerebral injury compared to the control group. This ancillary observational study investigated the effect of cerebral oximetry on electrocortical activity assessed by amplitude‐integrated electroencephalography (aEEG).

**Methods:**

aEEG from three centres participating in the COSGOD III trial was evaluated for the total maturation score (TMS) and its four individual component scores, the visual background pattern and sleep–wake cycling.

**Results:**

We included 147 preterm infants with a mean gestational age of 28.1 weeks and a mean birth weight of 1020 g: 70 in the NIRS group, 77 in the control group.

We found significantly higher total maturation scores, more mature sleep–wake cycles, and mature background patterns in the NIRS group compared to the control group.

**Conclusion:**

In the present cohort, NIRS‐guided oxygen delivery combined with dedicated treatment guidelines during the immediate transition after birth showed an impact on electrocortical activity in the neonatal period. Whether this improvement in maturational aspects of the aEEG is also associated with better outcome needs to be evaluated in the long term.

**Abbreviations**
aEEG: amplitude‐integrated electroencephalography
crSO_2_
: cerebral tissue oxygen saturation
NIRS: near infrared spectroscopy

**Summary**

- Oxygen delivery guided by cerebral near infrared spectroscopy (NIRS) during transition after birth leads to a non‐significant increase in survival without cerebral injury in preterm infants.
- NIRS‐guided oxygen delivery combined with dedicated treatment guidelines during the immediate transition after birth showed an impact on electrocortical activity in the neonatal period.
- Further research should investigate whether this improvement in maturational aspects of electrocortical activity is also associated with a better outcome.

## 1 Introduction

Premature birth increases the likelihood of neurodevelopmental impairment, largely attributed to a higher incidence of neonatal morbidities during neonatal period [1]. Hypoxia has been identified as a major factor in the development of brain injury during fetal‐to‐neonatal transition at birth. In the delivery room, monitoring of the neonatal condition is primarily confined to pulse oximetry, but pulse oximetry alone seems to be insufficient to identify preterm infants at risk for cerebral injury. A recent study group found out that only 23% of the included infants born before 32 weeks of gestational age achieved the transcutaneous oxygen saturation targets within 5 min after birth, a factor associated with adverse outcome [2]. Another study showed that incorporating continuous monitoring of cerebral regional tissue oxygen saturation (crSO_2_) alongside standard pulse oximetry during transition resulted in a 55% relative reduction of cerebral hypoxia burden among preterm infants [3]. In recent decades, there has been a significant increase in the importance of clinical monitoring of brain activity in preterm infants [4]. The amplitude‐integrated electroencephalogram (aEEG) has been established as a tool to monitor electrocortical activity in preterm infants in the last years [4, 5]. Both, aEEG and near infrared spectroscopy (NIRS) monitoring are feasible and of interest during the immediate postnatal transition [6, 7]. Abnormal cerebral activity patterns were detected in compromised neonates requiring resuscitation [8].

In a multicentre, randomised phase three clinical trial (COSGOD III) designed to assess NIRS‐guided oxygen delivery in preterm neonates during the immediate transition after birth, a non‐significant increase of 4.3% in survival without cerebral injury was detected in the NIRS group compared to the control group [9].

This ancillary observational study aimed to investigate whether electrocortical signals differ in very preterm infants according to the group allocation of the COSGOD III trial. We hypothesised that in preterm infants additional crSO_2_ guided oxygen delivery compared to routine care alone improves aEEG signals in the immediate fetal‐to‐neonatal transition.

## 2 Patients and Methods

### 2.1 Study Population and Study Design

In this retrospective observational study, neonates included in the prospective randomised‐controlled COSGOD III multicentre trial, conducted between October 2017 and February 2022, were eligible. The study protocol and the primary outcome of the COSGOD III trial have been published [9, 10]. Infants who were born at one of the three centres that participated in the COSGOD III trial (11 centres) with available data on aEEG (three out of 11 centres: Division of Neonatology, Department of Paediatrics and Adolescent Medicine, Medical University of Graz, Austria; Division of Neonatology, Paediatric Intensive Care and Neuropaediatrics, Department of Paediatrics Medical University of Vienna, Austria; and Department of Paediatrics II, Medical University of Innsbruck, Austria) were included in the present study. Centres with no available data on aEEG were excluded from analyses a priori. A total of 166 preterm infants (Graz *n* = 33, Innsbruck *n* = 65, Vienna *n* = 68) had aEEG recordings at the predefined time points and thus were eligible for the study. Nineteen infants were excluded for quality reasons. For the final analysis, 147 infants were included. For this ancillary study, aEEG signals were analysed and compared between the NIRS group and the control group of the COSGOD III trial.

### 2.2 COSGOD III Trial

The COSGOD III trial consisted of premature infants born below 32 weeks of gestational age. Infants were randomised in the three participating centres of the present analysis before delivery and allocated to the NIRS group or to control group. During the first 15 min after birth, all infants received continuous measurement of cerebral oxygenation. Resuscitation was conducted according to the local guidelines respectively the latest “Resuscitation Consensus guidelines” [11]. In the NIRS group, crSO_2_ monitoring was visible for the neonatologists. If peripheral oxygen saturation was between the normal limits, supportive measures were changed if crSO_2_ was below the 10th or above the 90th centile. The control group was treated only according to peripheral oxygen saturation limits as the crSO_2_ measurement was not visible to the resuscitation team. A detailed description of the COSGOD III trial has been published previously [9].

### 2.3 Patient Characteristics

Neonatal and perinatal data were collected during the hospital stay as described previously [12]. Gestational age was calculated according to obstetrical ultrasound or using the modified Ballard score. A premature rupture of membranes was diagnosed when it occurred more than 24 h before birth. Infants with a birth weight below the 10th centile for age and sex of the Fenton growth chart were classified as small for gestational age [13].

### 2.4 aEEG Recording and Assessment Details

The aEEG was recorded using the BrainZ instruments BRM 3 monitor (Natus Medical Inc., San Carlos, California, USA), the CFM Olympic BrainZ Monitor (Natus Medical Inc., San Carlos, California, USA) or the Unique CFM (Inspiration Health Care Group, Croydon, UK). All infants born below 32 weeks gestational age received aEEG monitoring after transition during the first 72 h after birth and then weekly from Week 1 to 4 for at least 3 h each. Electrode placement was set at the C3, P3, C4, and P4 positions according to the 10–20 international system of electrode placement. The cross‐cerebral signal was computed using the parietal electrodes (P3, P4). aEEG traces with artefacts, traces that were recorded under sedation, and traces with an impedance above 10 kΩ were excluded to maintain high quality. Also, recordings of less than 1 h were excluded. The assessment was done at several predefined evaluation points (0–6 h after birth, 6–12 h 18–24 h, 30–36 h, 42–48 h, 54–60 h, 66–72 h and at Week 1, 2, 3 and 4). Evaluation was performed by one investigator blinded to clinical data. In case of doubt, the aEEG was discussed with another investigator until consensus was reached. Both investigators have more than 10 years of experience in aEEG assessment.

All recordings were analysed according to the total maturation score (TMS) which was described by Burdjalov et al., consisting of four individual component scores: continuity, cycling, lower border of the amplitude, and bandwidth span All individual component scores were then summed up to the TMS, which consists of a maximum of 13 points. Higher TMS describe normal changes in aEEG associated with increasing maturation [14]. Furthermore, we assessed the visual background pattern (continuous, discontinuous, burst suppression, low voltage, flat trace) and sleep–wake cycling according to the presence of sleep–wake cycles, maturity of the sleep–wake cycles (none, immature sleep–wake cycles, incomplete sleep–wake cycles and mature sleep–wake cycles) and the number of sleep–wake cycles per hour [15, 16].

### 2.5 Statistical Analysis

Statistical analysis was performed using SAS 9.4 (2016 by SAS Institute Inc., Cary, NC, USA.). Data are shown as numbers (frequencies, %), median with interquartile range (IQR). For comparison of neonatal characteristics, maternal and fetal causes for preterm birth, and interventions during the first 15 min after birth and the first 24 h after birth between NIRS group and control group chi‐square test or Fisher's exact test were used for categorical variables and Mann–Whitney *U* test for metric variables. For the comparison of outcome variables between groups generalised linear models (probability distribution: binomial; link function: log; random effect centre) were used and relative risk (RR) with 95% CI were estimated. TMS was compared between groups using Mann–Whitney *U* test. Individual component scores, visual background pattern and sleep–wake cycles per hour were compared between groups using chi‐square test or Fisher's exact test. Results were regarded as statistically significant when *p* < 0.05.

### 2.6 Ethical Considerations

The trial was approved by the human research ethics committee at each participating site: Graz: institutional ethical board of the Medical University of Graz (reference number: 28–456 ex 15/16); Vienna: institutional review board of the Medical University Vienna (reference number: 1823/2017); Innsbruck: institutional ethical board of the Medical University of Innsbruck (reference number: 1048/2017).

## 3 Results

### 3.1 Study Population

Out of 277 eligible neonates included in the COSGOD III trial in the three centres, a total of 166 preterm infants (Graz *n* = 33, Innsbruck *n* = 65, Vienna *n* = 68) had aEEG recordings at the predefined time points. Nineteen infants were excluded for quality reasons. For the final analysis, 147 infants were included, with a median (range) gestational age of 28.1 (23.1–31.9) weeks and a median birth weight of 1020 (378–2220) grams. Seventy infants were randomised to the NIRS group and 77 infants to the control group.

We found no significant differences in neonatal characteristics and maternal/fetal causes for preterm birth between the groups (Table 1). In the outcome measurements, infants in the NIRS group had a lower rate of culture‐proven sepsis (*p* = 0.005), a lower rate of retinopathy of prematurity grade ≥ 2 (*p* = 0.030) and a higher rate of necrotizing enterocolitis (*p* < 0.001) compared to the control group (Table 2).

**TABLE 1:** Patient characteristics, interventions and outcome parameters of study participants.

(a) Neonatal characteristics, maternal and fetal causes for preterm birth			
Neonatal characteristics	NIRS group	Control group	*p* value
	*n*=70	*n*=77	
Gestational age, weeks, median (IQR)	28.3 (26.9–30.4)	27.4 (26.00–29.60)	0.091
Gestational age < 28weeks, *n* (%)	28 (40.0)	41 (53.3)	0.108
Birth weight, grams, median (IQR)	1100 (850–1370)	970 (810–1250)	0.343
Male, *n* (%)	41 (58.6)	43 (55.8)	0.739
Umbilical artery pH, median (IQR)	7.33 (7.28–7.38)	7.34 (7.30–7.40)	0.381
Apgar 1, median (IQR)	8.0 (6.0–8.0)	8.0 (6.0–8.0)	0.604
Apgar 5, median (IQR)	9.0 (8.0–9.0)	9.0 (8.0–9.0)	0.576
Apgar 10, median (IQR)	9.0 (9.0–9.0)	9.0 (9.0–9.0)	0.310
Maternal causes for preterm birth			
Antepartum bleeding, *n* (%)	6 (8.6)	8 (10.4)	0.708
Chorioamnionitis, *n* (%)	12 (17.1)	23 (29.9)	0.070
Premature rupture of membranes, *n* (%)	25 (35.7)	28 (36.4)	0.935
Preeclampsia, *n* (%)	14 (20.0)	10 (13.0)	0.251
Gestational diabetes, *n* (%)	2 (2.9)	2 (2.6)	1.000
Others, *n* (%)	11 (15.7)	22 (28.6)	0.062
Fetal causes for preterm birth			
Intrauterine growth restriction, *n* (%)	10 (14.3)	8 (10.4)	0.472
Fetal bradycardia, *n* (%)	12 (17.1)	6 (7.8)	0.084
Pathological doppler sonography, *n* (%)	7 (9.1)	0.094	13 (18.6)
Multiples, *n* (%)	5 (7.1)	4 (5.2)	0.737
Others, *n* (%)	0 (‐)	1 (1.3)	1.000

(b) Interventions during the first 15 min after birth and the first 24 h after birth			
First 15 min after birth			
Supplemental oxygen, *n* (%)	70 (100.0)	76 (98.7)	1.000
No respiratory support, *n* (%)	0 (‐)	0 (‐)	
Mask continuous positive pressure, *n* (%)	30 (42.9)	36 (46.8)	
Mask positive pressure ventilation, *n* (%)	36 (51.4)	36 (46.8)	
Intubation, *n* (%)	4 (4.9)	5 (6.5)	
Chest compressions, *n* (%)	1 (1.4)	2 (2.6)	1.000
Caffeine, *n* (%)	15 (22.1)	27 (35.1)	0.085
Adrenaline, *n* (%)	1 (1.4)	0 (‐)	
Surfactant, *n* (%)	2 (2.9)	3 (3.9)	1.000
Volume, *n* (%)	2 (2.9)	0 (‐)	0.225
Others, *n* (%)	2 (2.9)	0 (‐)	0.225
First 24 h after birth			
Surfactant, *n* (%)	47 (67.1)	56 (72.7)	0.460
No respiratory support, *n* (%)	2 (2.9)	5 (6.5)	0.511
Non‐invasive ventilation, *n* (%)	54 (77.1)	54 (70.1)	
Mechanical ventilation, *n* (%)	14 (20.0)	18 (23.4)	

*Note:* Study group by outcome with *n* (%), median (IQR).

**TABLE 2:** Patient characteristics, interventions, and outcome parameters of study participants.

Outcome measurements	NIRS group	Control group	Relative risk (95% CI)	*p* value
Death and/or cerebral injury, *n* (%)	10 (14.3)	16 (20.8)	0.69 (0.32–1.44)	0.321
Death, *n* (%)	2 (2.9)	0 (−)	—	—
IVH any grade, *n* (%)	8 (11.4)	16 (20.8)	0.55 (0.29–1.06)	0.072
No IVH, *n* (%)	62 (88.6)	61 (79.2)		0.058
IVH I‐II, *n* (%)	5 (7.1)	11 (14.3)		
IVH III‐IV, *n* (%)	3 (4.3)	5 (6.5)		
Cystic PVL any grade, *n* (%)	2 (2.9)	0 (−)	—	—
No cystic PVL, *n* (%)	68 (97.1)	77 (100.0)		—
Cystic PVL II	0 (−)	0 (−)		
Cystic PVL III	2 (2.9)	0 (−)		
IRDS any grade, *n* (%)	64 (91.4)	70 (90.9)	1.01 (0.95–1.06)	0.830
No IRDS, *n* (%)	6 (8.6)	7 (9.1)		0.561
IRDS grade 1–2, *n* (%)	53 (75.7)	57 (74.0)		
IRDS grade 3–4, *n* (%)	11 (15.7)	13 (16.9)		
Culture proven sepsis, *n* (%)	26 (37.1)	38 (49.4)	0.75 (0.62–0.92)	**0.005**
NEC any grade, *n* (%)	5 (7.1)	2 (2.6)	2.75 (2.00–3.78)	**< 0.001**
BPD, *n* (%)	10 (14.3)	13 (16.9)	0.85 (0.54–1.33)	0.469
ROP grade ≥ 2, *n* (%)	9 (12.9)	17 (22.1)	0.58 (0.36–0.95)	**0.030**
PDA with interventions, *n* (%)	13 (18.6)	16 (20.8)	0.89 (0.76–1.05)	0.170

*Note:* Study group by outcome with *n* (%), median (IQR). Bold values indicate *p* < 0.05.

Abbreviations: BPD, bronchopulmonary dysplasia; IRDS, infant respiratory distress syndrome; IVH, intraventricular haemorrhage; NEC, necrotizing enterocolitis; PDA, patent ductus arteriosus; PVL, periventricular leukomalacia; ROP, retinopathy of prematurity.

### 3.2 Total Maturation Score and Individual Component Scores

**FIGURE 1:**
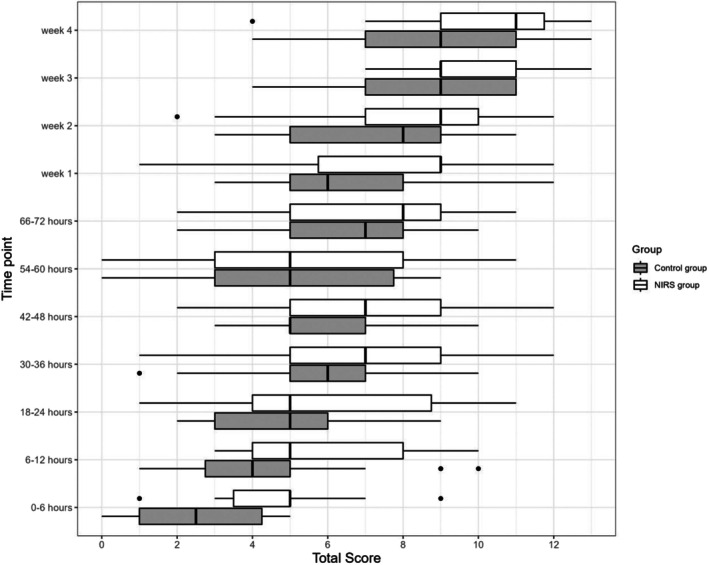
Total maturation score by study group. The continuous bold line shows the median. The x‐axis shows the total maturation score.

The aEEG TMS increased in both groups from Day 1 to Week 4. Pairwise comparisons showed significantly higher scores in the NIRS group at 0–6 h (*p* = 0.048), at 6–12 h (*p* = 0.024) and at Week 1 (*p* = 0.006) (Figure 1).

**FIGURE 2:**
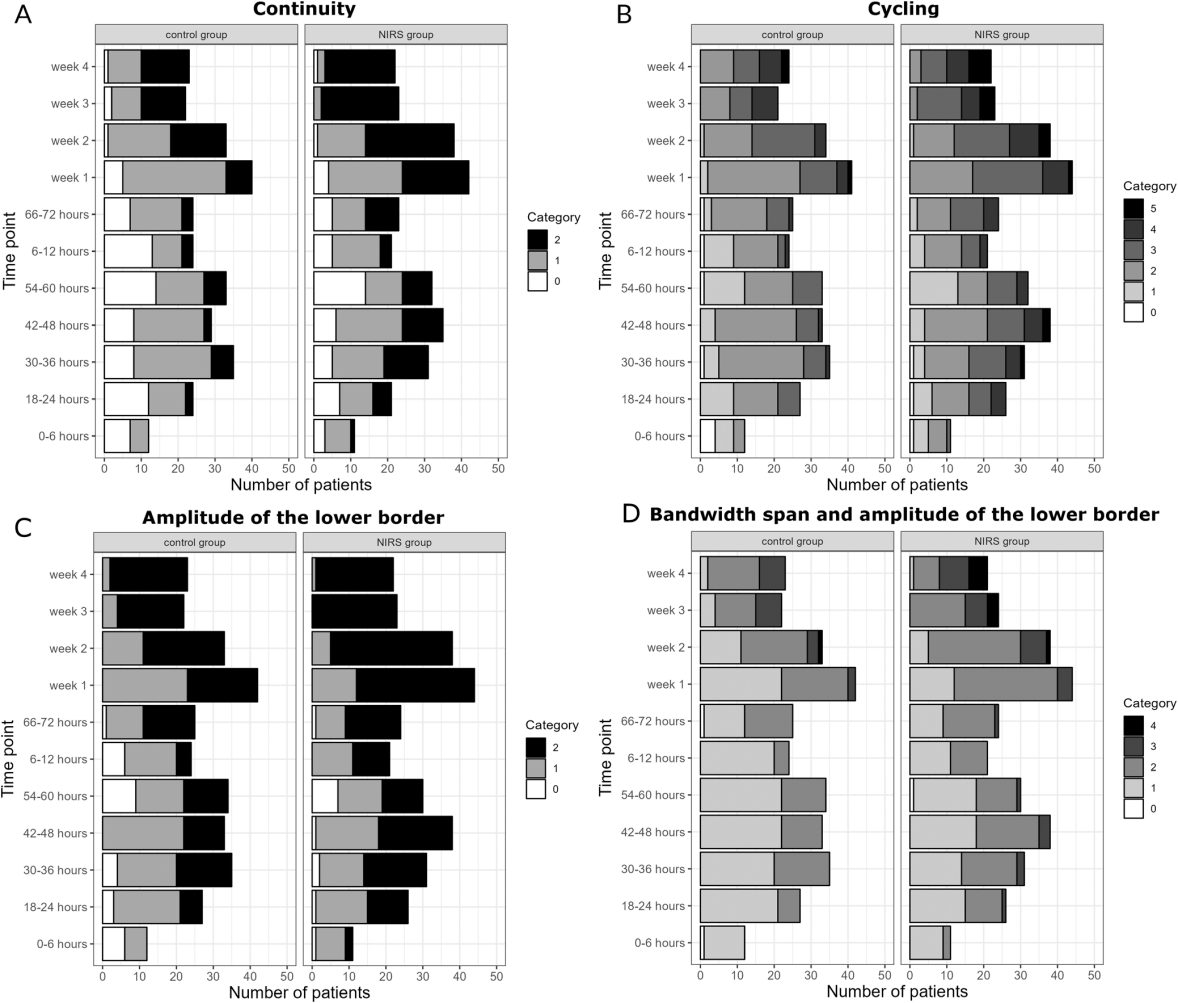
Individual component score by study group. The x‐axis shows the individual component score by group.

Continuity scores were higher in the NIRS group at 42–48 h (*p* = 0.049), at Week 1 (*p* = 0.048), at Week 3 (*p* = 0.014) and at Week 4 (*p* = 0.044) compared to the control group (Figure 2A). Cycling scores were higher in the NIRS group at Week 3 (*p* = 0.017). At the other evaluation points, no significant difference was found (Figure 2B). Infants from the NIRS group displayed significantly higher amplitude of the lower border scores at 6–12 h (*p* = 0.014), at Week 1 (*p* = 0.009), at Week 2 (*p* = 0.042), and at Week 3 (*p* = 0.049) (Figure 2C). The bandwidth span and amplitude of the lower border scores were higher in the NIRS group at 6–12 h (*p* = 0.025) and at Week 4 (*p* = 0.041) compared to the control group (Figure 2D).

### 3.3 Visual Background Pattern

**FIGURE 3:**
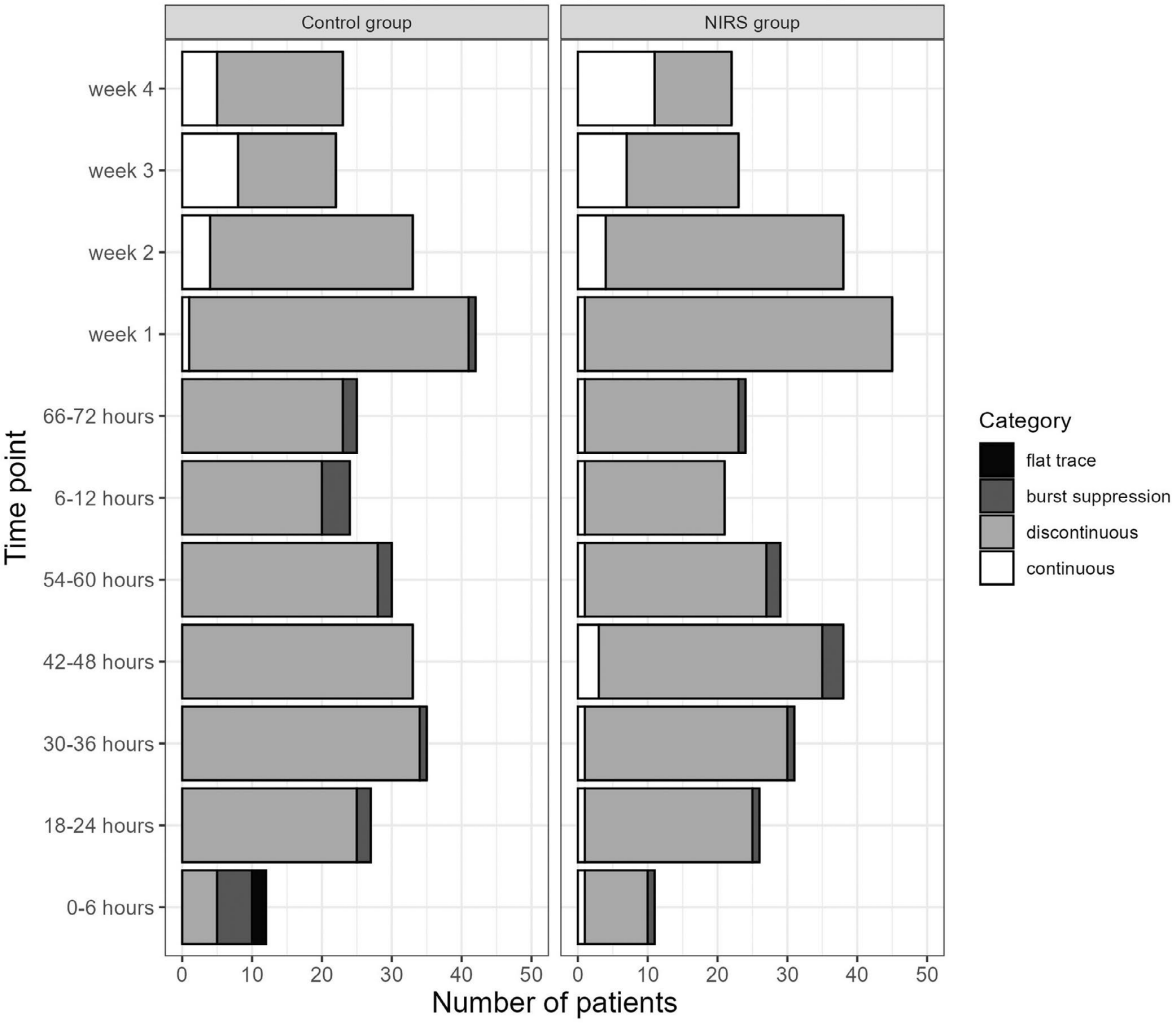
Visual background pattern by group. The x‐axis shows the number of patients.

For the visual background pattern, we found a higher percentage of continuous background pattern in the NIRS group at Week 4 (21.7% with continuous background in the control group (*n* = 5) versus 50% with continuous pattern in the NIRS group [*n* = 11], *p* = 0.048). At all other evaluation points, we found no difference between the two groups (Figure 3).

### 3.4 Sleep–Wake Cycling

**FIGURE 4:**
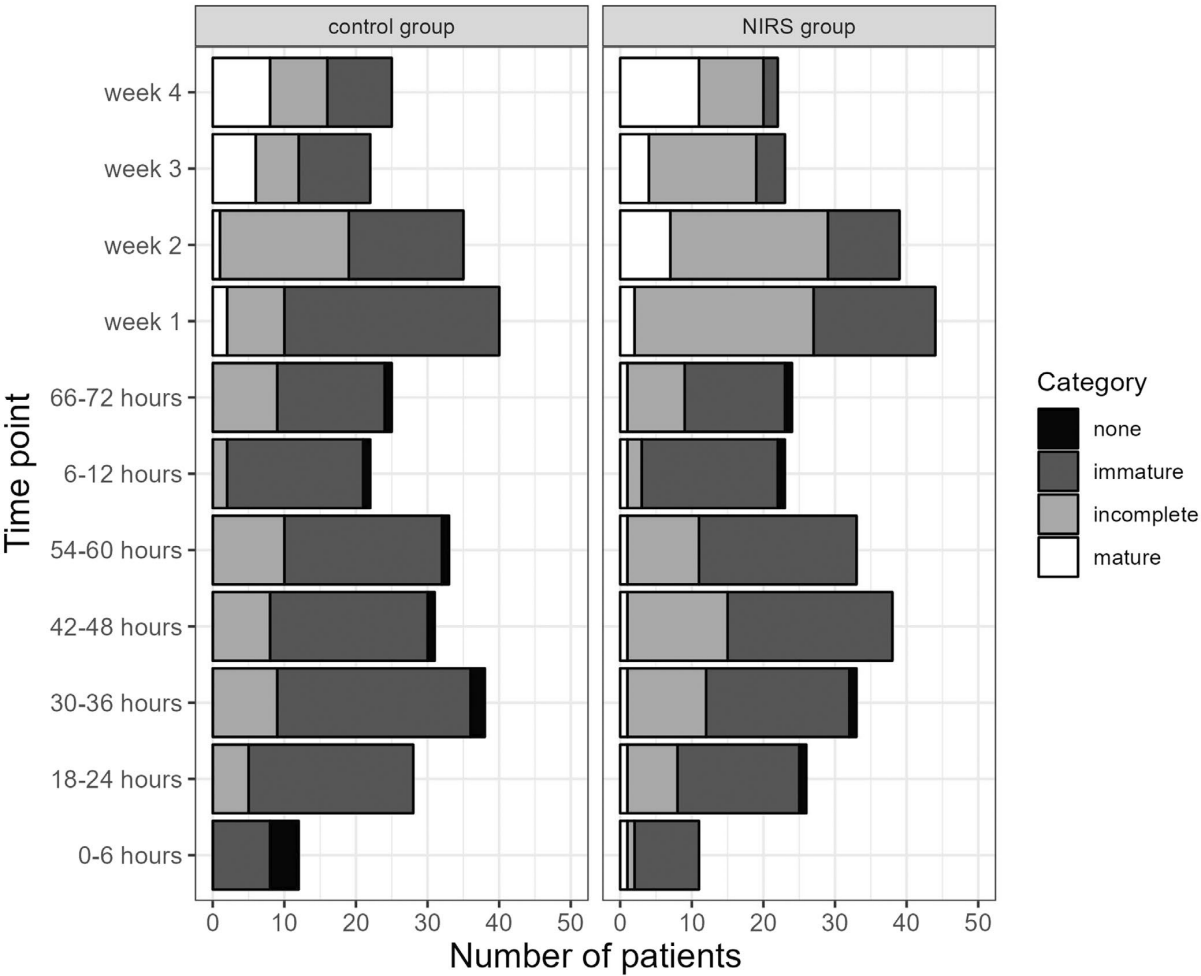
Sleep–wake cycle. The x‐axis shows the number of patients.

When comparing the presence and maturity of sleep–wake cycles per hour by group, we found significantly less immature sleep–wake cycles in the NIRS group at Week 1 (75% with immature sleep–wake cycles, 20% with incomplete sleep–wake cycles and 5% with mature sleep–wake cycles in the control group versus 38.6% with immature sleep–wake cycles, 56.8% with incomplete sleep–wake cycles and 4.5% with mature sleep–wake cycles in the NIRS group, *p* = 0.001), at Week 2 (45.7% with immature sleep–wake cycles, 51.4% with incomplete sleep–wake cycles and 2.9% with mature sleep–wake cycles in the control group versus 25.6% with immature sleep–wake cycles, 56.4% with incomplete sleep–wake cycles and 17.9% with mature sleep–wake cycles in the NIRS group, *p* = 0.041), and at Week 3 (45.5% with immature sleep–wake cycles, 27.3% with incomplete sleep–wake cycles and 27.3% with mature sleep–wake cycles in the control group versus 17.4% with immature sleep–wake cycles, 65.2% with incomplete sleep–wake cycles and 17.4% with mature sleep–wake cycles in the NIRS group, *p* = 0.034). At the other evaluation points, significance was not reached (Figure 4).

## 4 Discussion

With this ancillary study to the COSGOD III trial we assessed a potential effect of NIRS‐guided oxygen delivery in preterm neonates during the immediate fetal‐to‐neonatal transition period on cerebral activity. The main finding of our study is that infants who received support due to crSO_2_ during neonatal transition, showed superior aEEG tracings compared to the control group. Whether this improvement in maturational aspects of the aEEG in the NIRS group is also associated with better outcome needs to be evaluated in the long‐term. This study adds new insights into effects of support and oxygen application during immediate transition on electrocortical activity. Since aEEG signals and cerebral activity differed between the groups, irrespectively of cerebral injury, crSO_2−_guided support and oxygen titration appeared to have beneficial effects not only on injury free survival but on brain function as well. Remarkably, the NIRS group showed significantly more mature tracings not only during the first aEEG after birth, which could be attributed to the desirable oxygen saturation in the brain during the transition period, but also at subsequent evaluation time points. These findings suggest a potential long‐term benefit of targeted cerebral oxygenation during transition, which warrants further investigation.

Monitoring of cerebral oxygenation during fetal‐to‐neonatal transition was shown to be feasible to detect hypoxia in neonates regardless of gestational age [17, 18]. Lower crSO_2_ values during immediate transition were found to be associated with a higher risk for cerebral haemorrhage in preterm infants [17]. In the primary outcome of the COSGOD III trial, a non‐significant increase of 4.3% in survival without cerebral injury was detected in the NIRS group compared to the control group [9]. This can be confirmed for the subgroup of this ancillary study as the number of patients with survival without brain injury was even 6.5% higher in the NIRS group. The number of patients suffering from intraventricular haemorrhage was also lower in the NIRS group (88.6% vs. 79.2%, *p* = 0.058). For years, oxygen has been administered to preterm infants during and after neonatal transition reluctantly to achieve given peripheral oxygen saturation targets [2]. With the advent of monitoring cerebral oxygenation during neonatal transition, a new opportunity has emerged to protect the developing preterm brain at its most vulnerable stage.

Combining NIRS and aEEG monitoring could offer new insights and potentially identify infants at risk for brain compromise. In 2011, ter Horst et al. demonstrated a strong correlation between electrocortical activity and oxygen consumption using NIRS and aEEG in the neonatal period. They concluded that a combination of high fractional tissue oxygen extraction and low electrocortical activity indicates low perfusion or oxygen delivery to the brain [19]. In our ancillary study to the COSGOD III trial, the NIRS group demonstrated more mature sleep–wake cycles and higher continuity scores. Pairwise comparisons of the aEEG total maturation score revealed significantly higher scores in the NIRS group compared to the control group at 0–6 h (*p* = 0.048) and at 6–12 h (*p* = 0.024). This could be explained by a study from Tataranno et al., showing an increase in oxygen extraction in preterm infants with increased early electro‐cerebral activity in the first hours of life [20]. The impact of cerebral oxygenation during immediate transition after birth on neurological outcome has been described previously [17, 21]. Baik et al. showed that neonates with cerebral haemorrhages displayed lower crSO_2_ values during transition, and Pansy et al. showed inferior general movements in infants with cerebral hypoxia. In 2013, Pichler et al. performed aEEG and NIRS monitoring during the first 10 min of life in neonates [8]. In that study, uncompromised neonates displayed higher minimum and maximum aEEG amplitudes and lower crSO_2_ compared to infants requiring resuscitation. This is in contrast to Tamussino et al., who demonstrated that infants with low cerebral activity during transition showed low crSO_2_ but increased cerebral oxygen extraction [22]. The present study is a refined derivative of the work by Pichler et al., and there are several differences regarding the study protocol that impede a direct comparison. First, the study population differs: while the 2013 study assessed infants born at or above 34 weeks of gestation, the present study included preterm infants born below 32 weeks of gestation. Second, before aEEG assessment during the first 10 min of life was included, whereas we provided aEEG tracings during the first 72 h of life, with weekly recordings until postnatal Week 4. The early and weekly assessments following the immediate fetal‐neonatal transition might be of importance with regard to outcome prediction [23, 24]. So far, data on outcome prediction is scarce: in neonates with hypoxic‐ischaemic encephalopathy, Goeral et al. demonstrated that combining aEEG and NIRS monitoring has a higher predictive value for short‐term outcomes [25]. It is crucial to explore whether this finding can be extrapolated to the population of preterm infants.

In this study we analysed aEEG by different parameters: TMS has been widely used to assess the maturity of the aEEG traces and to predict neurodevelopmental outcome [14, 26]. We also aimed to distinguish pathological and physiological patterns and found differences between the groups by analysing visual background patterns and sleep–wake cycles [15, 27, 28].

Future studies should investigate whether the beneficial effects observed on aEEG signals are transient or if they lead to a reduction in brain injury and ultimately contribute to favourable long‐term outcome in the NIRS group. A strength of our study is the large cohort comprising patients from three distinct neonatology centres, providing a highly representative patient population. Our study demonstrated the beneficial short‐term effects of NIRS‐guided support and oxygen therapy during immediate transition, regardless of adherence to local guidelines, on preterm infants.

In conclusion, NIRS‐guided respiratory support and oxygen administration during immediate transition has beneficial effects for preterm infants. Infants who received respiratory support and oxygen based on crSO_2_ exhibited higher aEEG scores, more mature background patterns, and more developed sleep–wake cycles compared to those managed solely by peripheral oxygen saturation limits. Future research should prioritise investigating the potential long‐term neurodevelopmental outcomes associated with these observations.

## Author Contributions

All authors contributed to the study conception and design. Material preparation, data collection and analysis were performed by C.S., M.H. and E.G. Statistics and graphics were done by A.A. The first draft of the manuscript was written by C.S. and E.G. All authors commented on previous versions of the manuscript. All authors read and approved the final manuscript.

## Consent

Written informed consent was obtained from the parents.

## Conflicts of Interest

The authors declare no conflicts of interest.

## Collaborators

Division of Neonatology, Department of Paediatrics, Medical University of Graz, Graz, Austria: Marlies Bruckner, Corinna Binder‐Heschl, Daniel Pfurtscheller, Johann Martensen, Nina Höller, Evelyn Ziehenberger, Lukas Mileder, Berndt Urlesberger, Bernhard Schwaberger; Comprehensive Center for Paediatrics, Department of Paediatrics and Adolescent Medicine, Division of Neonatology, Intensive Care and Neuropediatrics, Medical University of Vienna, Vienna, Austria: Julia Buchmayer, Angelika Berger (local principal investigator), Sigrid Baumgartner, Agnes Grill, Michaela Mayr, Judith Rittenschober‐Boehm, Michael Schneider; Department of Paediatrics II, Neonatology, Medical University of Innsbruck, Innsbruck, Austria: Peter Wöckinger, Anna Posod; Neonatal Intensive Care Unit, Department for Perinatology, Division of Gynaecology and Obstetrics, University Medical Centre Ljubljana, Slovenia: Tina Perme, Lilijana Kornhauser‐Cerar, Anja Marolt, Ana Dimnik, Vlasta Lubej Kurtovič; Infant Centre, University College Cork, Cork University Maternity Hospital, Cork, Ireland: Eugene M Dempsey, Christoph E Schwarz, Garvey Aisling, Jurate Panaviene, David Healy, Nahla Ahmed, Ita Herlihy; Department of Neonatology, University Children's Hospital of Tübingen, Germany: Laila Springer, Kerstin Gründler, Axel Franz; Neonatologia e Terapia Intensiva Neonatale (TIN) Ospedale dei Bambini “V.Buzzi,” Milano, Italia: Gianluca Lista, Ilaria Stucchi, Francesca Castoldi, Francesco Cavigioli; II Department of Neonatology, Neonatal Biophysical Monitoring and Cardiopulmonary Therapies Research Unit, Chair of Neonatology, Poznan University of Medical Sciences, Poznan, Poland: Tomasz Szczapa, Lukasz Karpinski. Ginekologiczno Położniczy Szpital Kliniczny Uniwersytetu Medycznego im. Karola Marcinkowskiego w Poznańiu, Poznań, Poland: Zuzanna Kozłowska, Marcin Minta, Zuzanna Owsiańska, Sonia Kahtan, Natalia Neumann‐Klimasińska, Karolina Wróbel, Agata Kubiaczyk, Katarzyna Kosik, Katarzyna Olek, Michalina Bugiera, Julita Porwolik, Agnieszka Basiukajć, Elzbieta Czapla, Wojciech Łukaszuk, Katarzyna Gryczka, Dobrochna Naskręcka, Jan Mazela, Marta Szymankiewicz‐Bręborowicz; Division of Neonatology and Paediatric Intensive Care Medicine, Center for Paediatrics and Adolescent Medicine, Medical Center—University of Freiburg, Faculty of Medicine, University of Freiburg, Freiburg, Germany: Hans Fuchs, Daniel Klotz, Jana Baumgartner; Institute for Maternal and Child Health, “IRCCS Burlo Garofolo,” Trieste, Italy: Jenny Bua, Jana Bembich, Laura Travan; Department of Paediatrics, University of Alberta, Edmonton, Alberta, Canada: Georg M Schmölzer, Brenda Law, Po‐Yin Cheung.

## Data Availability

The data that support the findings of this study are available from the corresponding author upon reasonable request.
